# Psycho-Trauma, Psychosocial Adjustment, and Symptomatic Post-Traumatic Stress Disorder among Internally Displaced Persons in Kaduna, Northwestern Nigeria

**DOI:** 10.3389/fpsyt.2014.00127

**Published:** 2014-09-12

**Authors:** Taiwo Lateef Sheikh, Abdulaziz Mohammed, Samuel Agunbiade, Joseph Ike, William N. Ebiti, Oluwatosin Adekeye

**Affiliations:** ^1^Clinical Services, Federal Neuropsychiatric Hospital, Kaduna, Nigeria; ^2^Department of Psychiatry, Ahmadu Bello University, Zaria, Nigeria

**Keywords:** post traumatic stress disorder, internally displaced persons, psychosocial adjustment, depression, psycho-trauma

## Abstract

**Background:** In April 2011, a post election violent conflict in Northern Nigeria led to resettlement of internally displaced persons (IDPs) in a camp in Kaduna, the worst affected state. We set out to determine prevalence and socio-demographic factors associated with post-traumatic stress disorder (PTSD) among IDPs. We also determined types of psycho-trauma experienced by the IDPs and their psychosocial adjustment.

**Methods:** Cross-sectional systematic random sampling was used to select 258 adults IDPs. We used Harvard trauma questionnaire to diagnose “symptomatic PTSD,” composite international diagnostic interview (CIDI) for diagnosis of depression, and communal trauma event inventory to determine exposure to psycho-trauma. We assessed social adjustment using social provision scale. Multiple logistic regression analysis was used to determine independent predictors of PTSD.

**Results:** Of the 258 IDPs, 109 (42.2%) had a diagnosis of PTSD, 204 (79.1%) had poor living conditions, and only 12 (4.7%) had poor social provision. The most frequent psycho-traumas were destruction of personal property (96.1%), been evacuated from their town (96%) and witnessing violence (88%). More than half (58%) of IDPs had experienced 11–15 of the 19 traumatic events. Independent predictors of PTSD among respondents were having a CIDI diagnosis of depression (adjusted odds ratios 3.5, 95% confidence interval 1.7–7.5; *p* = 0.001) and witnessing death of a family member (3.7, 1.2–11.5; *p* = 0.0259).

**Conclusion:** We concluded that exposure to psycho-trauma among IDPs in Kaduna led to post conflict PTSD. Death of a family member and co-morbid depression were independent predictors of PTSD among IDPs. Though their living condition was poor, the IDPs had good psychosocial adjustment. We recommended a structured psychosocial intervention among the IDP targeted at improving living condition and dealing with the psychological consequences of psycho-trauma.

## Introduction

In April 2011, a post election violent conflict in Northern Nigeria killed 800 and displaced 65,000 people over a period of 3 days (1). The majority of the fatalities (88%) were from Kaduna state of Nigeria, known for ethno-religious conflicts. A camp for the internally displaced persons (IDPs) was set up in Kaduna city, the capital of the state to cater for the medical and social needs of the IDPs. Despite the increase in communal conflicts in Northern Nigeria after the country returned to democratic rule in 1999 (2), only few studies had been conducted on possible development of post-traumatic stress disorder (PTSD) among the survivors of the violent conflicts. Obilom and Thacher (3) studied PTSD following ethno-religious conflict in Jos, a neighboring state to Kaduna. He found “witnessing or been victims of personal attacks,” “losing possessions,” and “burning of homes” as the leading psycho-trauma among the study population.

We set out to determine the prevalence of PTSD, and types of psycho-trauma experienced by the IDPs and their psychosocial adjustment. We also determined factors associated with PTSD.

## Materials and Methods

### Study setting

The study was conducted in Kaduna city, the capital of Kaduna state located in the Northwestern zone of Nigeria in March 2013. The IDP camp was located on the outskirt of the city in a transit camp originally built for Hajj pilgrims (Muslims traveling to Saudi Arabia for the holy pilgrimage). Over 2,500 IDPs were settled in the camp.

### Study design

We conducted a cross-sectional study among male and female IDPs aged ≥18 years. We defined IDPs as people living within the camp, who have been displaced from their original communities as a result of the violent conflict following the April 2011 elections in Nigeria. We excluded persons already diagnosed with a mental disorder prior to the post election conflict and those who refused consent.

### Sample size determination

A minimum sample size of 230 was calculated using the Leslie and Kish (4) formula for estimating sample size for cross-sectional study.

$$n=\frac{\left(Z\alpha^{2}pq\right)}{d^{2}}$$

where *n* = minimum sample size; *Z*α set at 5% significant level = 1.96; *p* = estimate of prevalence of arousal symptoms of PTSD among the IDPs in a similar study in Nigeria = 84% = 0.84 (3); *d* = level of precision (5%); *q* = 1 − *p*.

$$\begin{array}{l} n=\frac{\left(1.96^{2}\times 0.84\times 0.16\right)}{0.05^{2}}\\ n=206 \end{array}$$

Adjusting for non-response rate of 10%

$$=\frac{nr}{\left(r-1\right)}$$

where *n* = calculated sample size and *r* = 10

$$\begin{array}{l} =\frac{206\times 10}{10-1}\\ =228 \end{array}$$

minimum sample size = 228.

For the study, we sampled 258 IDPs.

### Sampling technique

We used systematic sampling technique to select the respondents for the study. A sampling frame was created from the line list of all the IDPs obtained from the welfare committee of the camp. We excluded IDPs who were <18 years of age. Of the 2,500 IDPs, 1,502 were included in the final sampling frame for the study. We divided the sampling frame by the study sample size to determine the sampling interval. The first IDP was selected from the sampling frame using a table of random numbers, and then using the sampling interval we selected subsequent respondents until the required sample size was achieved.

### Study instrument

We designed a questionnaire to measure the socio-demographic characteristics of IDPs and their living conditions, which was assessed by asking the following questions: availability of sleeping mat, private facility, toilets or latrine, sufficient food, and protection from animals and insects for individual IDPs. We also asked if the accommodations were cramped, if their health was good, and if they had enough organizational support. Conflict-related trauma was assessed with a shortened version of the communal traumatic events inventory used to study Bosnian refugees (5). We included only trauma events, the IDPs were likely to have experienced and respondents were to indicate “yes” or “no” depending on experience during the conflict. To measure the IDPs psychosocial adjustment, we adapted the social provision scale originally developed by Cutrona and Russell (6). Twelve of the 24 questions that captured the components of reassurance of worth, reliable alliance, and guidance were used for the study. We defined good psychosocial adjustment as answering “strongly agree” or “agree” to 8 of the 12 questions. We used the composite international diagnostic interview (CIDI) to generate diagnosis of depression among the respondents. CIDI is a highly structured clinical interview instrument derived from the National Institute of Mental Health (NIMH) diagnostic interview schedule (DIS) and the present state examination (PSE). The instrument has been proven to have cross-cultural validity (7) and the Hausa version was used in a community survey in Nigeria (8). Finally, we used the Harvard trauma questionnaire (HTQ) (9) designed by Harvard Program in Refugee Trauma, Massachusetts General Hospital for the diagnosis of symptomatic PTSD. The PTSD section consists of 16 questions based on the diagnostic criteria of the diagnostic and statistical manual for mental disorders fourth edition (DSM IV) (10). The questions were measured on a 5-point severity scale of 1–5. Scores for each respondent were summed up and divided by the number of items (16) to derive the score for each individual. Individuals with total score >2.5 were considered symptomatic for PTSD (9). The cut off score of 2.5 had been standardized for several version of HTQ (11–13) and the HTQ had been validated for use in displaced persons in several cross-cultural studies (14–16). The questionnaire was translated to Hausa the main language spoken in Northern Nigeria and back translated to English. The translation underwent detailed review by the study team and followed recommended guidelines (9, 17).

### Data collection and procedure

We recruited six data collectors who could speak both English and Hausa language fluently and were experienced in data collection from prior activities. They were trained for a period of 5 days on the use of the study questionnaire and interview techniques prior to the commencement of the study. Data collection took place over a period of 6 days and the average duration of each interview was 40 min. Five supervisors made up of psychiatrists and senior resident doctors supervised the data collectors.

### Data entry and analysis

Data were entered into Epi info 3.3.2, cleaned, and edited for inconsistencies before analysis. Descriptive statistics were used in summarizing the data, while analytical statistics were used to test for significant associations and predictors. We used frequencies, proportions, and tables to summarize our data, bivariate analysis and multiple logistic regressions were performed to identify factors associated with the occurrence of symptomatic PTSD. We dichotomized the outcome of HTQ based on a cut off score of 2.5 into those with PTSD and those without PTSD. The chi square test was used in determining statistically significant associations, while factors with *p* values <0.05 were included in the logistic regression model. Adjusted odds ratios (AORs) were determined with 95% confidence interval (CI) to identify predictors of PTSD.

### Ethical consideration

Ethical approval was granted by the health research ethical committee of the Federal Neuro-Psychiatric Hospital Kaduna before onset of the study. Participating IDPs were required to give an informed consent before inclusion in the study. All potential subjects were presented with a consent form, which described the type of study being done, the purpose of the study, and the subject’s rights as a participant in the study, including the right to confidentiality and the right to withdraw from the study. Names were not included in any of the findings and no financial inducement was given to any respondent. Two psychiatrists, four psychiatric nurses, a clinical psychologist, and a pharmacist provided medical advice if needed and at the end of the study those determined to have significant distress were referred to a specialist psychiatric hospital for management.

## Results

A total of 258 interviews were conducted. The mean age of the IDPs was 38.7 years (SD: 15.2 years) with a range of 18–95 years. Of the 258 IDPs, 134 (51.9%) were females, all (100%) practiced Islam, 53 (20.5%) were widowed and 68.2% were unemployed (Table 1).

**Table 1:** **Socio-demographic characteristics of IDPs^a^ by gender of respondents (*n* = 258)**.

Characteristics	*N*	(%)
**Sex**
Female	134	(51.9)
**Religion**
Islam	258	(100)
**Marital status**
Married	154	(59.7)
Widowed	53	(20.5)
Single	35	(13.6)
Divorced	11	(4.3)
Separate	5	(1.9)
**Level of education^b^**
Quranic	101	(39.3)
Primary	68	(26.5)
Secondary	50	(19.5)
None	31	(12.1)
Tertiary	7	(2.7)
**Employment status**
Unemployed	176	(68.2)
Employed	65	(25.2)
Student	2	(0.8)
Retired	3	(1.2)

*^a^IDPs, internally displaced persons*.

*^b^*n* = 257*.

Of the 258 respondents, 109 (42%) of them had a diagnosis of PTSD.

The most trauma event experienced by the IDPs (Table 2) were destruction of personal property (96.1%) and been evacuated from their town (96%). More than three-quarter had witnessed violence (88%), reported the death of a family member (87%), and suffered ill health (84%). More than two-thirds had witnessed torture (69%) and witnessed death of family member (68%). Only four (1.7) IDPs reported rape or sexual molestation. More than half (58%) of the IDPs had experienced 11–15 of the 19 traumatic events covered by the questionnaire.

**Table 2:** **Exposure of IDPs^a^ to traumatic events (*N* = 258)**.

Events	Number of persons experiencing event		
	Number	%	95% CI^b^
**Type of trauma event experienced**			
Destruction of personal property^c^	247	96.1	93.1–98.1
Evacuated from town	247	95.7	92.5–97.9
Lost property^b^	246	95.7	92.5–97.8
Separated from loved ones	239	92.6	88.7–95.5
Witness violence	227	88.0	83.4–91.7
Death of family member	224	86.8	82.1–90.7
Ill health^b^	215	83.7	78.6–88.0
Family beaten	180	69.8	63.8–75.3
Witness torture^b^	177	68.9	62.8–74.5
Witness death of family member^b^	175	68.1	62.0–73.7
Without shelter^b^	165	64.2	58.0–70.1
Stolen possession^b^	154	59.9	53.7–66.0
Shortage of clothing^b^	150	58.4	52.1–64.5
Shortage of medicine^b^	149	58.0	51.7–64.1
Physical injury	118	45.7	39.5–52.0
Beaten	101	39.1	33.2–45.4
Lack of food	100	38.8	32.8–45.0
Loved ones disappear	86	33.3	27.6–39.4
Rape or sexual molestation^d^	4	1.7	0.5–4.2
**Cumulative trauma events experienced**			
0–5	4	1.6	0.4–3.9
6–10	57	22.1	17.2–27.7
11–15	149	57.8	51.5–63.9
≥16	48	18.6	14.0–23.9

*^a^IDPs, internally displaced persons*.

*^b^CI, confidence interval*.

*^c^*n* = 257*.

*^d^*n* = 240*.

The exposure of female and male IDPs to 9 of the 19 traumatic events showed significant variations (Table 3). Of the IDPs that were beaten, only 27% were females [odd ratio (OR) 0.2, 95% CI 0.1–0.3; *p* < 0.000]. Among IDPs witnessing death, less than half (43%) were females (0.3, CI, 0.2–0.6; *p* < 0.000) and only 37% of females had physical injury (0.3, 0.2–0.6; *p* < 0.000). Among IDPs that had witnessed torture, 45% were females (0.4, 0.2–0.7; *p* < 0.001). Other traumatic events that showed significantly less exposure to females were lack of food (0.4, 0.3–0.7; *p* < 0.001), disappearance of loved ones (0.4, 0.2–0.7; *p* < 0.001), having personal belongings been stolen (0.5, 0.3–0.8; *p* = 0.003), and being without shelter (0.6, 0.4–1.1; *p* = 0.01). The only psycho-trauma with more females been exposed (54.5%) was ill health (2.0, 1.0–4.0; *p* = 0.05).

**Table 3:** **Types of trauma event experienced by IDP^a^ by gender**.

Events	Number of persons experiencing event					
Type of trauma event experienced	Female		Male		OR^b^ (95% CI^c^)	*p* Value
	*n*	%	*n*	%	
Beaten	27	26.7	74	73.3	0.2 (0.1–0.3)	<0.000
Witness death of family member	76	43.4	99	56.6	0.3 (0.2–0.6)	<0.000
Physical injury	44	37.3	74	62.7	0.3 (0.2–0.6)	<0.000
Witness torture	79	44.6	98	55.4	0.4 (0.2–0.7)	<0.001
Lack of food	39	39.0	61	61.0	0.4 (0.3–0.7)	<0.001
Loved ones disappear	32	37.2	54	62.8	0.4 (0.2–0.7)	<0.001
Stolen possession	68	44.2	86	55.8	0.5 (0.3–0.8)	0.003
Without shelter	79	47.9	86	52.1	0.6 (0.4–1.1)	0.01
Ill health	118	54.9	97	45.1	2.0 (1.0–4.0)	0.05
Lost property	131	53.3	115	46.7	3.0 (0.8–11.7)	0.09
Evacuated from town	124	51.0	121	49.0	0.4 (0.1–1.5)	0.2
Family beaten	89	49.4	91	50.6	0.7 (0.4–1.2)	0.2
Destruction of personal property	127	51.4	120	48.6	0.5 (0.1–1.8)	0.3
Shortage of clothing	74	49.3	76	50.7	0.8 (0.5–1.3)	0.4
Witness violence	116	51.1	111	48.9	0.8 (0.4–1.6)	0.5
Shortage of medicine	79	53.0	70	47.0	1.1 (0.7–1.9)	0.6
Separated from loved ones	125	52.3	114	47.7	1.2 (0.5–3.1)	0.7
Death of family member	117	52.2	107	47.8	1.1 (0.5–2.2)	0.8
Rape or sexual molestation	2	50.0	2	50.0	0.8 (0.1–5.7)	0.8

*^a^IDPs, internally displaced persons*.

*^b^OR, odds ratio*.

*^c^CI, confidence interval*.

Of the 258 IDPs, 109 (42.2%) had a diagnosis of PTSD, 204 (79.1%) had poor living conditions, but only 12 (4.7%) had poor social provision.

At bivariate analysis 6 of the 19 trauma events covered by this study were significantly associated with a diagnosis of symptomatic PTSD (Table 4). IDPs who had PTSD were 5 times more likely to have witnessed death of a family member (5.03, 1.9–13.5; *p* < 0.00), 3 times more likely to have been exposed to ≥16 trauma events (2.8, 1.5–5.3; *p* < 0.00), and twice more likely to have had their personal belonging stolen (2.0, 1.2–3.4; *p* < 0.01). Furthermore, IDPs with a diagnosis of PTSD were four times more likely to have a co-morbid diagnosis of depression (3.8, 1.9–7.7; *p* < 0.00). There were no socio-demographic associations of PTSD.

**Table 4:** **Factors associated with PTSD^a^ among IDPs**.

Characteristics	PTSD		No PTSD		OR^b^ (95% CI^c^)	*p* Value
	*n*	(%)	*n*	(%)	
**Death of family member**					**5.03 (1.9–13.5)**	**<0.00**
Yes	104	(95.4)	120	(80.5)
No	5	(4.6)	29	(19.5)	
Total	109	(100)	149	(100)	
**CIDI Depression**					**3.8 (1.9–7.7)**	**<0.00**
Yes	29	(26.6)	13	(8.7)
No	80	(73.4)	136	(91.3)	
Total	109	(100)	149	(100)	
**Exposed to ≥16 events**					**2.8 (1.5–5.3)**	**<0.00**
Yes	30	(27.5)	18	(12.1)
No	79	(72.5)	131	(87.9)	
Total	109	(100)	149	(100)	
**Stolen possession**					**2.0 (1.2–3.4)**	**<0.01**
Yes	75	(69.4)	79	(53.0)
No	33	(30.6)	70	(47.0)	
Total	108	(100)	149	(100)	
**Witness violence**					**2.9 (1.2–6.8)**	**0.02**
Yes	102	(93.6)	125	(83.9)
No	7	(6.4)	24	(16.1)	
Total	109	(100)	149	(100)	
**Ill health**					**2.4 (1.1–4.9)**	**0.02**
Yes	94	(89.9)	117	(79.1)
No	11	(10.1)	31	(20.9)	
Total	109	(100)	148	(100)	
**Witness death**					**2.0 (1.1–3.3)**	**0.02**
Yes	82	(75.9)	93	(62.4)
No	26	(24.1)	56	(37.6)	
Total	108	(100)	149	(100)	
**Shortage of clothing**					**1.7 (1.0–2.8)**	**0.04**
Yes	71	(65,7)	79	(53.0)
No	37	(34.3)	70	(47.0)	
Total	108	(100)	149	(100)	

*^a^PTSD, post traumatic stress disorder*.

*^b^OR, odds ratio*.

*^c^CI, confidence interval*.

We performed multiple logistic regression analysis on all the eight factors that were significantly associated with PTSD at bivariate analysis (Table 5). Of the eight factors, having a CIDI diagnosis of depression (AOR 3.5, 1.7–7.5; *p* < 0.001) and experiencing death of a family member (3.7, 1.2–11.5) remained as independent predictors of PTSD among the IDPs.

**Table 5:** **Independent predictors of PTSD^a^ among IDPs on multivariate analysis**.

Term	PTSD^a^ (*N* = 254) AOR	95% CI^b^		*p* Value
CIDI depression (yes/no)	*3.5271*	*1.6641*	*7.4757*	*0.001*
Death of family (yes/no)	*3.6679*	*1.1694*	*11.5049*	*0.0259*
Ill health (yes/no)	1.677	0.7309	3.8478	0.2225
Shortage of clothing (yes/no)	1.1906	0.6646	2.1332	0.5575
Stolen possessions (yes/no)	1.5012	0.8289	2.7189	0.1801
Exposed to ≥16 trauma events (yes/no)	1.7406	0.8129	3.7269	0.1537
Witness violence (yes/no)	1.9378	0.6921	5.4255	0.2079
Witnessed death (yes/no)	1.4134	0.7474	2.6727	0.2872

*^a^PTSD, post traumatic stress disorder*.

*^b^CI, confidence interval*.

*Italic fonts represent data that were of statistical significance*.

## Discussion

In this study, destruction of personal property, being evacuated from town, witnessing violence, reported death of a family member, and suffering ill health were the most frequent psycho-trauma experienced by the IDPs. The violent nature of the conflict was demonstrated by more than half of the IDPs experiencing 11–15 psycho-traumatic events during the conflict. The level of exposure of IDPs to psycho-traumatic events such as witnessing death of a relative, possessions being stolen, and witnessing violence were much higher among this IDPs compared to a similar study done in Northern Nigeria (3). Rape and sexual molestation violence appeared not to have been widely used in this conflict. The prevalence of rape and sexual molestation was similar to that reported in a study in Bosnia (18), but it is much lower than that reported in a study of IDPs in Northern Uganda (16). We found females to be significantly less exposed to psycho-trauma, which is consistent with the findings of higher exposure of men to psycho-trauma during conflicts reported in Northern Uganda. Due to the less exposure of females to psycho-trauma we expected a lower prevalence of PTSD in the females but this was not the finding. There was no significant difference in the PTSD prevalence between the genders. This could suggest that women have a higher risk of developing PTSD due to lower threshold from exposure to psycho-trauma compared to men. This finding has been demonstrated in previous studies (16).

The prevalence of 42% for PTSD is high when compared with findings from similar studies among Guatemalan refugees with PTSD prevalence of 11.2% and prevalence of 5.6% among Bosnian refugees. The prevalence of PTSD among IDPs found in this study is similar to the 41% found by Obilom et al. in Northern Nigeria in different set of IDPs displaced by a different conflict but lower than the 54% found among Ugandan IDPs in Northern Uganda.

We found death of a family member and co-morbid depression to be the only independent predictors of PTSD among the IDPs. Several studies have found association between PTSD and depression (19, 20). Depressive disorders can occur independently after exposure to psycho-trauma and a previous depressive disorder is a risk factor for developing PTSD. We did not find significant association between socio-demographic characteristics and PTSD despite the high degree of unemployment among the IDPs. Despite the apparent poor social provisions at the camp, the IDPs did not report significant poor psychosocial adjustment, this may be explained by the lack of social amenities that is characteristic of communities were the IDPs were living prior to the conflict. The communities lack portable water, poor environmental sanitation, and poor housing conditions. Therefore, the camp condition may not differ significantly from their pre conflict environmental conditions and thus have little effects on IDPs psychosocial adjustment.

The findings of this study are subjected to the following limitations. This study was conducted 2 years post conflict therefore recall bias was possible but the nature of the psycho-trauma made it possible that the IDPs recollected their experience easily. The response to sensitive traumatic events, especially rape and molestation may have been under reported, especially among the females. We studied only IDPs living in the camps and excluded those that were staying with relatives or have moved out of the camps for other reasons. Some researchers have questioned the validity of PTSD diagnosis in non-western setting (21), but the instruments used for this study have been widely used and validated for different cultural settings.

## Conclusion

We concluded that exposure to psycho-trauma among IDPs living in Hajj camp in Kaduna, Northern Nigeria led to post conflict PTSD. Though males were more exposed to psycho-trauma there was no difference in gender PTSD prevalence. Death of a family member and co-morbid depression were independent predictors of PTSD among the IDPs. Though there living condition was poor, the IDPs had good psychosocial adjustment. PTSD among the IDPs can be predicted by IDPs who witnessed the death of a family member or had a co-morbid diagnosis of depression. We recommended a structured psychosocial intervention among the IDP targeted at improving living conditions and dealing with the consequences of psycho-trauma to the state government. We also advocated for the setting up of weekly mental health clinic in the camp to help those IDPs with mental distress.

## Conflict of Interest Statement

The authors declare that the research was conducted in the absence of any commercial or financial relationships that could be construed as a potential conflict of interest.
